# NK cell-derived IL-10 is critical for DC-NK cell dialogue at the maternal-fetal interface

**DOI:** 10.1038/s41598-017-02333-8

**Published:** 2017-05-19

**Authors:** Sandra M. Blois, Nancy Freitag, Irene Tirado-González, Shi-Bin Cheng, Markus M. Heimesaat, Stefan Bereswill, Matthias Rose, Melanie L. Conrad, Gabriela Barrientos, Surendra Sharma

**Affiliations:** 1 Charité-Center for Internal Medicine and Dermatology, Division of General Internal and Psychosomatic Medicine, Reproductive Medicine Research Group, Charité-Universitätsmedizin Berlin, Berlin, Germany; 2 Department of Pediatrics, Women and Infants Hospital-Warren Alpert Medical School of Brown University, Providence, RI USA; 3 Department of Microbiology and Hygiene, Charité-Universitätsmedizin Berlin, Berlin, Germany; 4 Laboratorio de Medicina Experimental, Hospital Alemán - Consejo Nacional de Investigaciones Científicas y Técnicas, Buenos Aires, Argentina

## Abstract

DC-NK cell interactions are thought to influence the development of maternal tolerance and de novo angiogenesis during early gestation. However, it is unclear which mechanism ensures the cooperative dialogue between DC and NK cells at the feto-maternal interface. In this article, we show that uterine NK cells are the key source of IL-10 that is required to regulate DC phenotype and pregnancy success. Upon *in vivo* expansion of DC during early gestation, NK cells expressed increased levels of IL-10. Exogenous administration of IL-10 was sufficient to overcome early pregnancy failure in dams treated to achieve simultaneous DC expansion and NK cell depletion. Remarkably, DC expansion in IL-10^−/−^ dams provoked pregnancy loss, which could be abrogated by the adoptive transfer of IL-10^+/+^ NK cells and not by IL-10^−/−^ NK cells. Furthermore, the IL-10 expressing NK cells markedly enhanced angiogenic responses and placental development in DC expanded IL-10^−/−^ dams. Thus, the capacity of NK cells to secrete IL-10 plays a unique role facilitating the DC-NK cell dialogue during the establishment of a healthy gestation.

## Introduction

Maternal immune tolerance allows development and normal term delivery of the semiallogeneic fetus, although the decidualized uterus contains highly specialized immune cells^1^. In this regard, the highly choreographed immune system at the maternal-fetal interface regulates placentation and trophoblast invasion, resulting in pregnancy-compatible *de novo* angiogenesis. The cooperative dialogue between dendritic cells (DC) and natural killer (NK) cells is an important aspect of the temporally co-ordinated uterine immune milieu and has attracted a great deal of interest^2–4^. We and others have shown that decidual DC influence both the growth and functional properties of uterine NK (uNK) cells during early gestation^5^. Indeed, *in vitro* studies have shown that co-culturing decidual CD56^+^ NK cells with immature DC stimulates their proliferation and activation^6^. It has also been reported that uterine DC improve their tolerogenic capacity to induce regulatory T cells (Tregs) upon interaction with uNK cells^7^, and reciprocally, promote proliferation and differentiation of IL-10 producing NK cells^8^. Secretion of IL-10, in turn, is likely an important signal modulating this crosstalk as studies in IL10^−/−^ mice have shown an increased susceptibility to inflammatory insults promoting immunogenic activation of DC (i.e., low doses of LPS or other TLR ligands), which cause pregnancy demise due to excessive infiltration and cytotoxic activation of uNK cells^9–11^. In addition, Prins *et al*. recently described that unstable Foxp3^+^ Treg and altered DC are associated with LPS-induced fetal loss in IL10^−/−^ dams^12^. Thus, IL-10 seems to be a critical mediator during DC-NK cell dialogue and may be important to fine-tune maternal innate and adaptive immune responses at the feto-maternal interface.

NK cells and DC act cooperatively to regulate non-immune processes vital to the maintenance of pregnancy. In this context, we have shown *in vitro* that trophoblasts promote decidual cell transformation but only in the presence of signals derived from DC and NK cells^2^. Additionally, DC-depleted implantation sites are characterized by decreased levels of IL-15 resulting in reduced numbers of NK cells^13^. These NK cells also have impaired differentiation failing to produce the levels of IFN-γ necessary for spiral artery remodeling^5^. Though it is evident that a synchronized DC-NK cell crosstalk is necessary for successful pregnancy, the molecular basis for such cellular interaction remains poorly understood.

In an effort to elucidate the crosstalk between DC and NK cells during early gestation, we recently described an *in vivo* model where altered DC/NK cell abundance at the maternal interface leads to abortion^4^. Furthermore, we observed that NK cells are revealed to be critical for shaping immunomodulatory functions of decidual DC during early gestation. In the absence of uNK cells pregnancy failed due to unbalanced production of anti-angiogenic signals and increased expression of inflammatory genes by decidual DC^4^. In the current study, we present data that support the notion that the capacity of NK cells to secrete IL-10 is critical for the maintenance of a healthy gestation. Specifically, our results confirm that uNK cell-derived IL-10 represents a distinct mechanism to modulate decidual DC functions compatible with a successful gestation, influencing the angiogenesis process associated with early gestation and placental development.

## Results

### IL-10 restores pregnancy compatible DC functions modulated by NK cells

**Figure 1:**
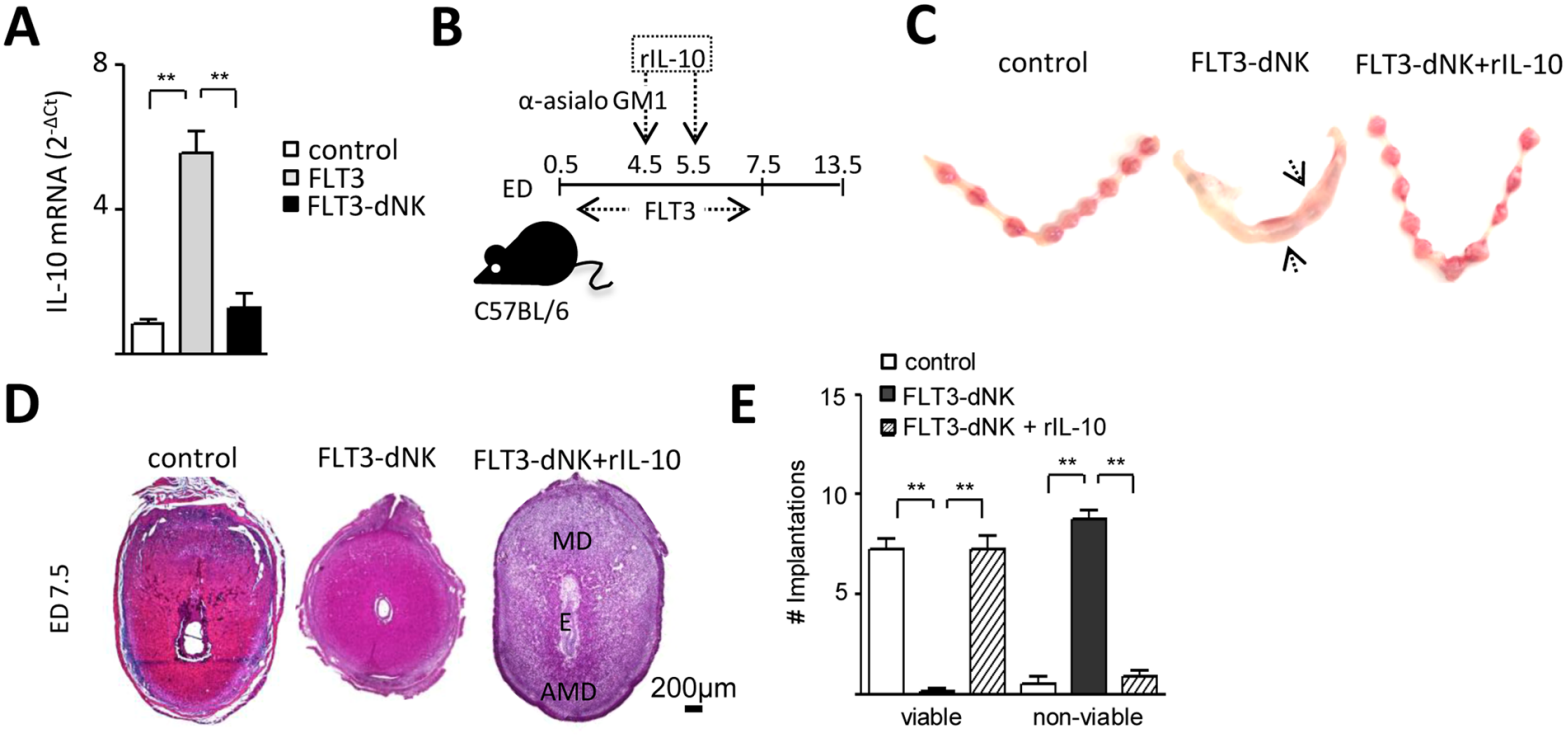
IL-10 rescues pregnancy failure upon NK cell depletion in FLT3-treated dams. (**A**) IL-10 expression in implantation sites from control, FLT3 treated (FLT3) and FLT3 treated- NK cell depleted (FLT3-dNK) pregnant female mice on ED7.5, as analysed by qRT-PCR. **p < *0.01*; as assessed by ANOVA, Tukey multiple comparisons test. (**B**) The scheme illustrates the experimental design for DC expansion and NK cell depletion during early pregnancy. Upon plug detection; female mice were injected daily with 10 μg FLT3 i.p. + a single anti-asialo GM1 injection on ED4.5 to analyze effects of NK cell depletion upon expansion of DC (FLT3-dNK mice). Recombinant IL-10 (rIL-10) was administrated on ED4.5 and ED5.5. (**C**) Macroscopical appearance of the uterus and implantations registered during E7.5. The pictures show the normal phenotype of control and FLT3-dNK + rIL-10 implantations in contrast to that observed in FLT3-dNK mice, which exhibit completely resorbed embryos. (**D**) Microscopical assessment of H&E stained implantation site sections revealed abnormalities in the decidual architecture of dams treated with FLT3 and anti-asialo GM1 (FLT3-dNK), with no distinguishable mesometrial (MD) and antimesometrial pole (AMD) and no signs of developed embryos on ED7.5. Scale: 200 μm. (**E**) Number of viable and non-viable implantations from dams treated with FTL3 and anti-asialo GM1 (FLT3-dNK) and dams treated also with rIL-10 on ED7.5. Data shown are mean ± SEM derived from six to eight mice per group. **p < *0.001* as analyzed by the Mann-Whitney U test.

**Figure 2:**
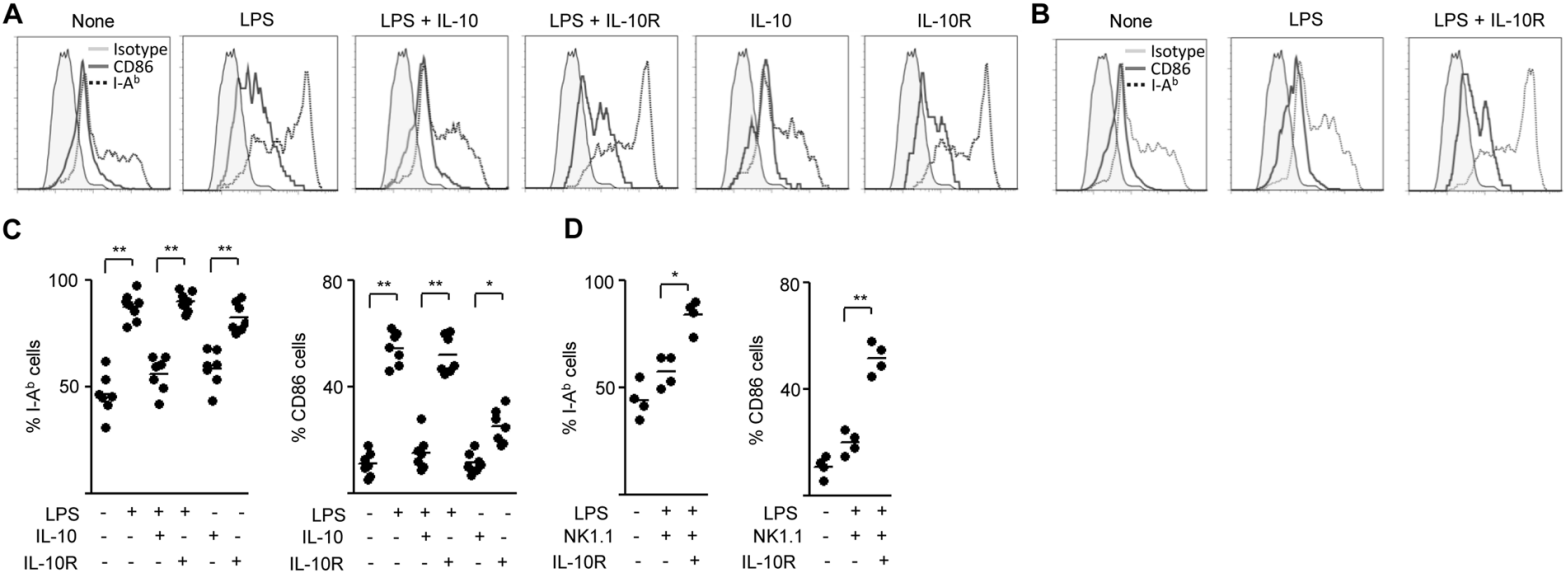
IL-10 rescues pregnancy failure upon NK cell depletion in FLT3-treated dams modulating decidual DC phenotype. (**A**) Representative flow cytometric histograms showing I-A^b^ and CD86 staining in gated decidual CD11c^+^ cells exposed to 1 μg/ml LPS in the presence or absence of IL-10 (10 ng/ml) or IL-10R (10 μg/ml). (**B**) IL-10^−/−^ CD11c^+^ cells were co-cultured with IL-10^+/+^ NK1.1^+^ cells and exposed to 1 μg/ml LPS in the presence or absence of IL-10R (10 μg/ml), and flow cytometric histograms are shown, which are representative of four mice. (**C**) Summary data showing the percentages of I-A^b^ and CD86 in gated decidual CD11c^+^ cells compared to control cultures with or without LPS, IL-10 or IL-10R. Data shown are mean derived from seven mice per treatment. (**D**) Summary data showing the percentage of I-A^b^ and CD86 in gated decidual IL-10^−/−^ CD11c^+^ cells compared co-cultures with IL-10^+/+^ NK1.1^+^ cells with or without IL-10R. Data shown are mean derived from four mice per treatment. (**C**,**D**) * and ** denote *p* < *0.05* and *p* < *0.01* respectively, as analysed by the Tukey’s test.

Pregnancy loss provoked by NK cell depletion following DC expansion is associated with increased expression of genes involved in immune activation, inflammation and unbalanced production of antiangiogenic signals^4, 14^. To identify mediators involved in the NK cell-mediated effect on DC during early gestation, we first examined decidual IL-10 expression in control, DC expanded (FLT3) and DC expanded - NK cell depleted (FLT3-dNK) gravid mice. As shown in Fig. 1A, when DC are expanded, IL-10 expression increased significantly in implantation sites compared to control mice and the effect was abrogated in FLT3-dNK mice. Next, we administered exogenous IL-10 to FLT3-dNK mice on ED 4.5 and 5.5 (Fig. 1B; n = 6–8 mice/group). Notably, the administration of exogenous IL-10 rescued fetal loss associated with the NK cell depletion in FTL3 mice (Fig. 1C–E; n = 6–8 mice/group). Given the well-defined role of IL-10 in DC biology, we sought to determine whether IL-10 was able to influence the phenotypic characteristics of decidual DC. As shown in Fig. 2A,C, *in vitro* LPS activation of decidual DC resulted in enhanced expression of MHC class II and CD86 expression which was significantly down-regulated in the presence of IL-10. However, this effect is abrogated if decidual DC are incubated with an IL-10 activity neutralizing antibody (IL-10R). In order to determine whether IL-10 released by NK cells exerts the effect on the phenotype of decidual DC activated by LPS, we co-cultured IL-10^−/−^ decidual DC with IL-10^+/+^ NK cells in the presence of LPS. As shown in Fig. 2B, IL-10^+/+^ NK cells were able to replicate the effect of IL-10 on the MHC-II and CD86 expression on decidual IL-10^−/−^ DC. This effect was inhibited by a neutralising IL-10 antibody (Fig. 2B,D).

Further examination revealed that IL-10 treatment in FLT3-dNK dams can enhance VEGF bioavailability *in vivo* by reducing sFlt-1 levels as depicted in Table 1. This is consistent with pro-angiogenic properties of this cytokine during gestation^15^. Next, we used quantitative real-time PCR to examine decidual tissue for the expression of VEGF and Flt-1 mRNA. Consistent with the results in Table 1, we found up-regulation of VEGF mRNA expression and down-regulation of Flt-1 mRNA upon IL-10 treatment *in vivo*. These results strongly suggest that IL-10 is able to modulate decidual DC functions and phenotype, and contributes to the angiogenesis process associated with early gestation when NK cells are temporarily depleted.

**Table 1:** IL-10 contributes to the angiogenesis process associated with early gestation when NK cells are temporarily depleted.

Parameter	FLT3-dNK	FLT3-dNK + rIL-10
sFlt-1 (pg/ml)	4038 ± 424*	1938 ± 337
VEGF mRNA (2^−ΔCT^)	1.31 ± 0.18	8.88 ± 1.83*
Flt-1 mRNA (2^−ΔCT^)	3.52 ± 0.35*	1.42 ± 0.37

Data are means ± SEM (n = 6–8/group). Serum sFlt-1 levels are shown as mean values analyzed by ELISA on ED7.5 and decidual VEGF and Flt-1 expression on ED7.5, as analysed by qPCR. *p < *0.05*, using two-tailed *t-*test.

### DC expansion in IL-10^−/−^ mice provokes pregnancy loss

**Figure 3:**
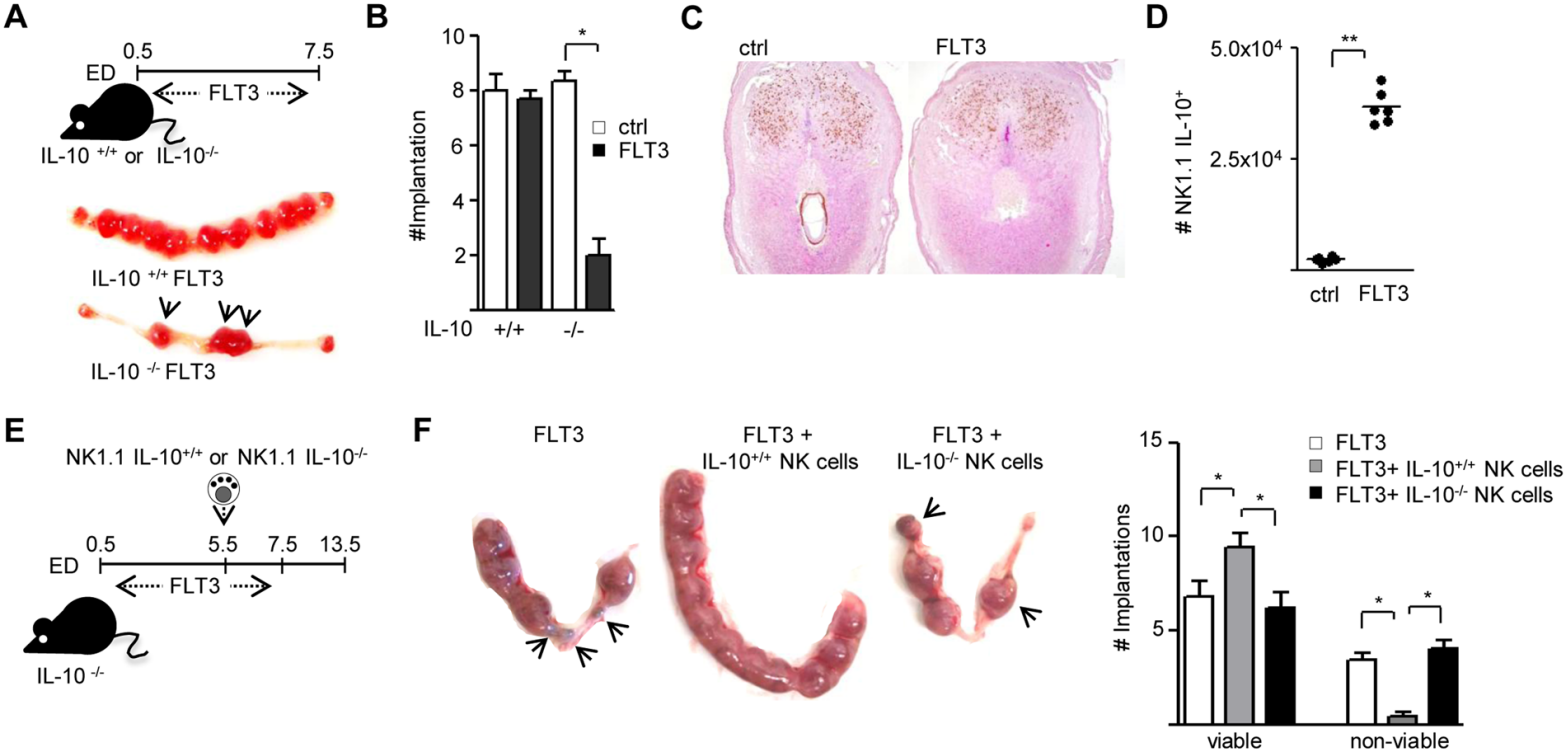
IL-10^+^ NK cells promote fetal survival in FLT3 treated IL-10^−/−^ mice. (**A**) Top panel: Protocol scheme for FLT3 treatment in female mice during early gestation. C57BL/6 females (IL-10^+/+^ or IL-10^−/−^) were treated with FLT3 from ED0.5 to ED7.5, as described in Methods. Bottom panel: Representative images of the macroscopical appearance of the uterus and implantation sites registered during ED7.5. The pictures show the normal phenotype of IL-10^+/+^ implantation sites in contrast to that observed in IL-10^−/−^ mice, which exhibit less implantations. (**B**) Number of implantations observed after FLT3L treatment in IL-10^+/+^ and IL-10^−/−^ dams on ED7.5. (**C**) Left panel: Representative images showing DBA-PAS staining of whole implantation sections harvested on ED7.5 from IL-10^+/+^ mice in which DC had been expanded with FLT3. (**D**) Absolute numbers of IL-10 expressing NK1.1 cells in control and FLT3 treated IL-10^+/+^ dams. In figures (**B**) and (**D**) data are depicted as the mean ± SEM derived from six to eight mice per group. *p < *0.05* and **p < *0.001* analyzed by the Mann-Whitney U test. (**E**) Experimental design for adoptive transfer of IL-10^+/+^ NK cells during early pregnancy. Upon plug detection; IL-10^−/−^ mice were injected daily with 10 μg FLT3 i.p. from ED0.5 to ED7.5. IL-10^+/+^ or IL-10^−/−^ NK1.1 cells were then adoptively transferred to IL-10^−/−^ mice upon FLT3 treatment on ED5.5. (**F**) Left panel: macroscopical appearance of the uterus on E13.5. The pictures show the normal phenotype of FLT3 treated IL-10^−/−^ dams transferred with IL-10^+/+^ NK cells in contrast to that observed in FLT3 treated IL-10^−/−^ dams transferred or not with IL-10^−/−^ NK cells mice, which exhibit resorbed fetuses; Right panel: Number of viable and non-viable implantations from FLT3 treated IL-10^−/−^ dams transferred or not with IL-10^+/+^ or IL-10^−/−^ NK cells observed on ED13.5. Data shown are mean ± SEM derived from six mice per group. *p < *0.05* analyzed by the Tukey’s test.

IL-10 is an anti-inflammatory cytokine that displays potent activity to suppress the antigen presentation capacity of APCs^16^ and protects against LPS- and CpG-induced pregnancy complications^10, 12^. It is then feasible that an increased immunogenic potential of decidual DC due to IL-10 deficiency could also contribute to pregnancy failure in FLT3-dNK mice. To evaluate further whether IL-10 is important during the DC-NK cell cross talk, we examined the effect of DC expansion during early gestation in IL-10^−/−^ mice and their wild type counterparts (Fig. 3A, top panel). As shown in Fig. 3A (bottom panel), we observed that compared to eDC IL-10^+/+^ dams, expansion of DC in IL-10^−/−^ mice induced a significant reduction of the number of implantation sites (Fig. 3B; n = 6 mice/group). Further analysis revealed that expansion of DC during early gestation is accompanied by an increased abundance of IL-10^+^ NK cells, while total NK cells numbers remained similar (Fig. 3C,D). Thus, soluble IL-10 released by NK cells influences the dialogue with DC during early gestation.

### IL-10^+/+^, but not IL-10^−/−^, NK cells rescue pregnancy loss in eDC IL-10^−/−^ mice

To test the function of IL-10^+/+^ NK cells from wild type dams, we adoptively transferred this subset of NK cells into FLT3-IL-10^−/−^ pregnant females on ED5.5 (Fig. 3E; n = 6 mice/group). The number of non-viable implantations was significantly reduced upon transfer of IL-10^+/+^ NK cells suggesting that IL-10 facilitates the DC-NK cell cross talk, ensuring a healthy pregnancy outcome (Fig. 3F). Of note, adoptive transfer of IL-10^−/−^ NK cells into FLT3-IL-10^−/−^ dams did not improve pregnancy outcome (Fig. 3F).

**Figure 4:**
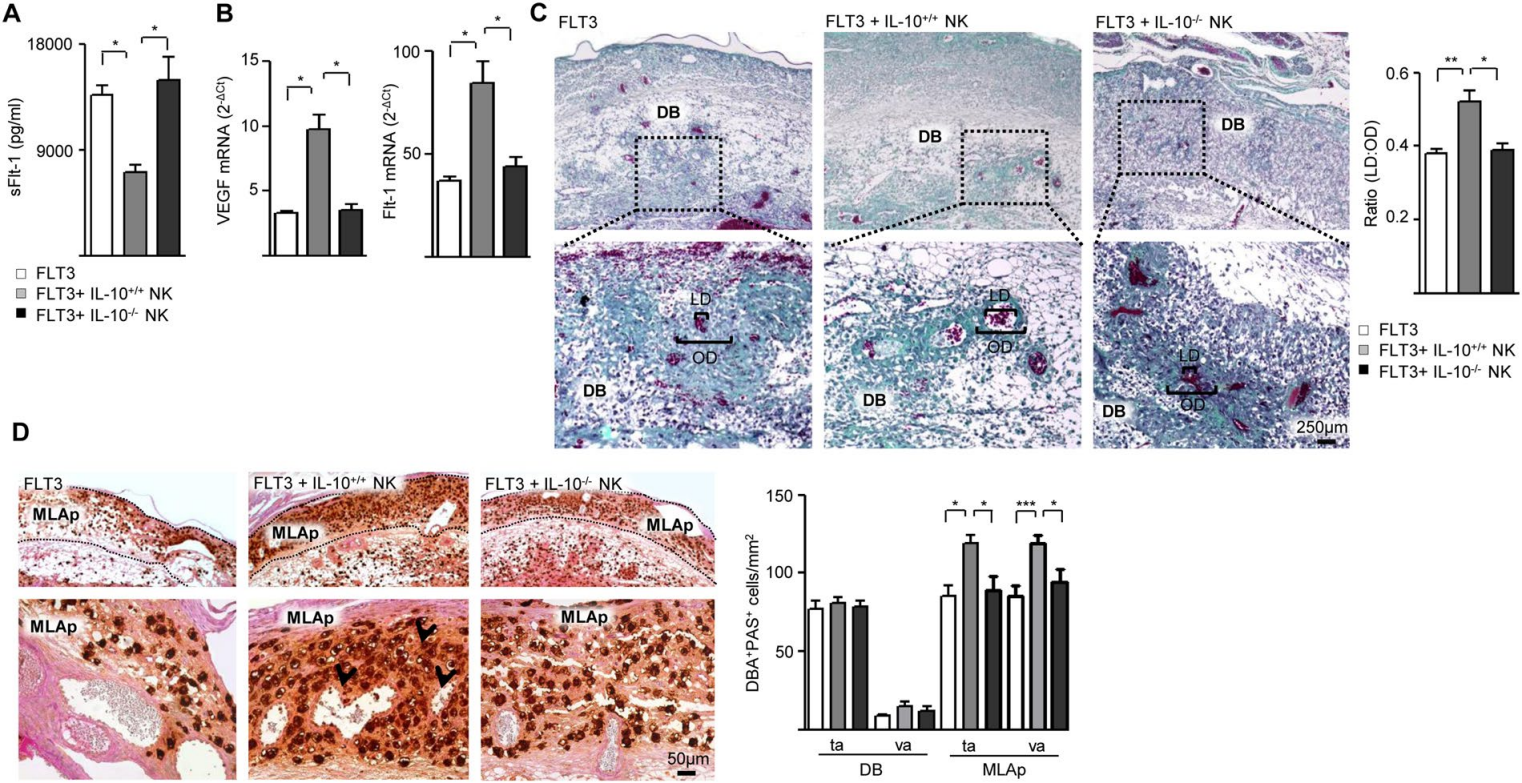
IL-10^+^ NK cells regulate placentation in FLT3 treated IL-10^−/−^ mice. (**A**) Serum levels of sFlt-1 analyzed by ELISA derived from FLT3 treated IL-10^−/−^ dams transferred or not with IL-10^+^ NK cells on ED13.5. (**B**) Tissue VEGF and Flt-1 expression on ED13.5, as analysed by qPCR. Data shown are mean values ± SEM derived from five to six mice per group each analyzed in duplicate. *p < *0.05* using the Tukey’s test. (**C**) Left panel: Representative trichrome-stained decidua sections of FLT3 treated IL-10^−/−^ dams adoptively transferred or not with IL-10^+^ NK cells show the maternal vessels in the decidua basalis on ED13.5. Bar = 250 μm. Right panel: Quantification of the inner lumen-to-outer diameter ratio (LD/OD) of the spiral arterial walls recorded on ED13.5. A total of 24 vessels were analysed and derived from six dams per group. (**D**) Left panel: Representative photomicrographs of dual DBA-PAS stained sections of implantation sites from ED13.5 are presented. Bar = 50 μm; Right panel: The number of uterine NK cells associated with spiral arteries was scored. When NK cells were intramural, they were scored as vascular associated (va; arrows). Eight to ten sections/implantation sites were scored. *p < *0.05* and **p < *0.001* analyzed by the Tukey’s test. Abbreviations: MLAp: mesometrial lymphoid aggregate of pregnancy; DB: decidua basalis; va: vascular associated; ta: tissue associated.

**Figure 5:**
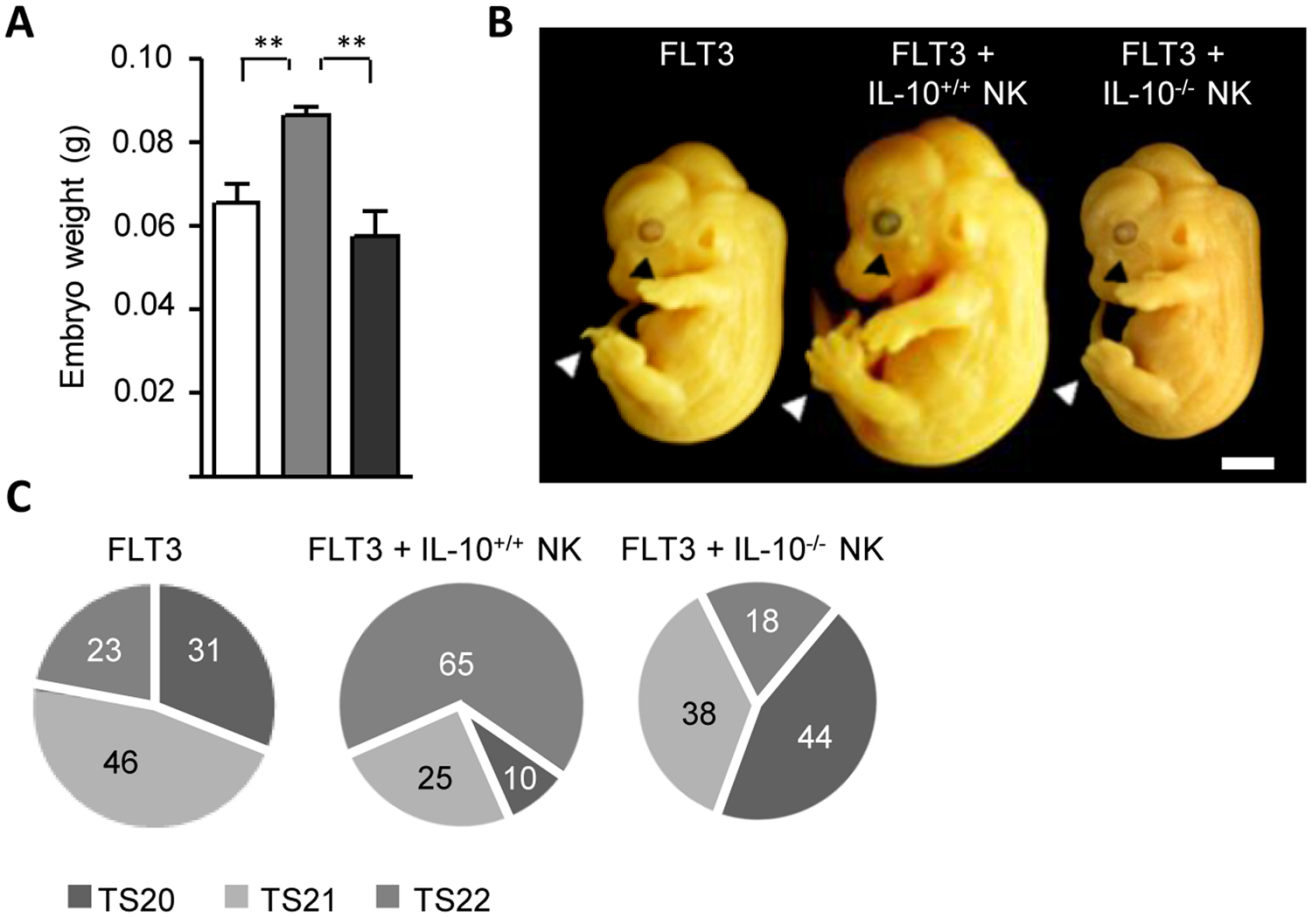
IL-10^+^ NK cells abrogate fetal growth restriction in FLT3 treated IL-10^−/−^ mice. (**A**) Mean body weights registered in the offspring following FLT3 treatment in IL-10^−/−^ female mice with or without IL-10^+/+^ NK cells adoptive transfer during early pregnancy. Data shown are mean ± SEM derived from 25–30 fetuses per group. (**B**) Representative images of fetuses obtained on ED13.5 from FLT3 treated IL-10^−/−^ female mice with IL-10^+/+^ or IL-10^−/−^ NK cells adoptive transfer; Bar = 0.25 cm. White arrows denote separated fingers on the toes and black arrows indicate the visible hair follicles. (**C**) Distribution of Theiler stage (TS) of fetal development on ED13.5. Twenty five to thirty fetuses derived from FLT3 treated in IL-10^−/−^ female mice with IL-10^+/+^ or IL-10^−/−^ NK cells adoptive transfer were scored. The distribution of TS is shown using a pie chart. The percentages of fetuses corresponded to TS20, TS21 and TS22 are indicated for each pie slice. **p < *0.01* analyzed by the Tukey’s test.

Changes in the immune-angiogenesis process associated with implantation have been mostly linked with the modulation of placental physiology during early and mid pregnancy^17, 18^. In particular, the maternal spiral arteries expand during mid gestation in order to increase blood flow to the placental bed^19^, and this adaptation process appears to be sensitive to alterations in uNK cells and DC abundance^20^. Thus, our next aim was to compare the progression of gestation upon adoptive transfer of IL-10^+/+^ NK cells focusing on the placentation period. Figure 4A shows that FLT3-IL-10^−/−^ pregnant females had elevated circulating levels of sFlt-1 compared to their counterparts adoptively transferred with IL-10^+/+^ NK cells. Next, we used quantitative real-time PCR to examine the tissue expression of VEGF and Flt-1 mRNA on E13.5. As shown in Fig. 4B up-regulation of VEGF mRNA expression and down-regulation of Flt-1 mRNA was observed only upon adoptive transfer of IL-10^+/+^ NK cells. Furthermore, Trichrome staining showed that only FLT3-IL-10^−/−^ mice adoptively transferred with IL-10^+/+^ NK cells had healthy dilated, thin-walled arteries in the central, proximal region of the decidua basalis at ED13.5 (Fig. 4C). Analysis of PAS^+^DBA^+^ granulated NK cells, which facilitate dilation of spiral arteries^18^, showed similar abundance and vascular- associated distribution pattern in the decidua basalis in both groups (Fig. 4D). In the mesometrial lymphoid aggregate of pregnancy (MLAp), however, FLT3-IL-10^−/−^ mice adoptively transferred with IL-10^+/+^ NK cells showed increased numbers of both vascular- associated and tissue associated NK cells (Fig. 4D). Thus, the adoptive transference of IL-10^+/+^ NK cells into FLT3-IL-10^−/−^ dams not only reduced fetal loss rates, but created a better environment for placental development. Consistent with this notion, we observed that fetuses carried by FLT3-IL-10^−/−^ dams appeared to be smaller compared to FLT3-IL-10^−/−^ females adoptively transferred with IL-10^+/+^ NK cells on ED13.5 (Fig. 5A). When fetal development was assessed according to the Theiler Stage (TS) fetus units carried by FLT3-IL-10^−/−^ dams adoptively transferred with IL-10^+/+^ NK cells depicted a typical TS22 with the deep indentations between the toes, individual fingers visible, a forward-turned pinna and hair follicles present in the pectoral, pelvic and trunk region (Fig. 5B). However, by ED13.5 most of the fetuses carried by FLT3-IL-10^−/−^ female mice showed an immature development (TS20/21) characterized by low indentations between the toes, the pinna at a right angle to the head and the absence of hair follicles (Fig. 5B,C).

## Discussion

It has been suggested that the dialogue between DC and NK cells during early gestation plays a critical role for pregnancy maintenance^2, 4^. With unique features the DC-NK cell liaison modulates not only immune properties of both cell types but also vital processes during gestation such as decidualization and angiogenesis^2, 4^. The findings reported in this article have intriguing implications for understanding the DC-NK cell liaison and highlighted a critical role of IL-10 as soluble mediator involved directly and indirectly in the orchestration of the embryo maternal dialogue during early gestation.

We showed here that during decidual DC expansion in the absence of a ‘danger’ signal, NK cells restrain DC immunogenic activation by enhanced IL-10 expression. Reciprocally, DC were shown to play an important role in NK cell homeostasis by enhancing proliferation and survival through IL-15 transpresentation^21^. Consistent with this notion, we have found that IL-10^+^ NK cells are upregulated after decidual DC expansion in comparison to untreated gravid mice. This upregulation response appears to be critical for pregnancy maintenance, as this and our previous study demonstrated that only in the presence of NK cells, DC expansion is not detrimental to pregnancy. Together, our data point out that although FLT3 treatment may promote the immunogenic functions of decidual DC, a pregnancy compatible DC phenotype is re-established as a result of their crosstalk with decidual NK cells. Further, the central role of IL-10 in mediating the dialogue between DC and NK cells is evidenced *in vivo* by the improved pregnancy outcomes observed in FLT3-dNK dams following administration of IL-10. There is evidence that IL-10 is not essential for the growth and development of the fetus in mice but rather it plays an important role to inhibit excessive inflammation^9, 10^. Thus, IL-10^−/−^ mice are more susceptible to immunogenic insults during pregnancy compared to wild type mice, such as treatment with low doses of LPS^12^ and CpG (a TLR 9 agonist)^9^ or FLT3 mediated DC expansion as shown in the present study. Indeed, our *ex vivo* experiments show that decidual DC respond to LPS in a different pattern when an intact IL-10 axis is present, consistent with recent studies showing an increased expression of MHC II and CD80 in the uterine DC pool of LPS treated pregnant IL-10^−/−^ mice^12^. Notably, our co-culture experiments showed that the *in vitro* modulation of DC phenotype by IL-10 could be replicated solely by the presence of wild type NK1.1^+^ cells, further reinforcing the concept that NK cell derived IL-10 is an important mediator quenching excessive inflammation during gestation.

By showing that pregnancy rates in DC expanded and simultaneously NK cell depleted females were improved upon treatment with exogenous IL-10, our study is the first to identify IL-10 as a soluble factor by which NK cells modulate DC functions in the uterus. However, our findings also point out that physical interactions play an important role during the DC-NK cell liaison, since normal numbers of implantations upon FLT3 treatment require not only an intact NK cell pool but also a normal IL-10 axis (as in IL-10^+/+^ animals). Unlike wild type animals, IL-10^−/−^ dams were more susceptible to fetal loss upon FLT3 treatment but pregnancy progression could be restored by adoptive transfer of IL-10^+/+^ NK1.1 cells. Knowing that IL-10 is an anti-inflammatory cytokine that displays potent abilities to suppress the antigen presentation capacity of APCs^16^ and protects against LPS-, CpG-induced pregnancy complications^10, 12^, it is feasible that pregnancy failure both in the FLT3-dNK model and in FLT3 treated IL-10^−/−^ mice results from an increased immunogenic potential of DC due to poor or ablated IL-10 secretion respectively. In these models, DC expanded in the absence of NK cell derived IL-10 most likely fail to promote a functional pregnancy protective T cell response as noted in LPS-challenged IL-10^−/−^ pregnant mice, which exhibit an unstable Treg pool associated with immunogenic activation of DC^12^. As for physical NK-DC interactions, though their importance for the modulation of NK survival and maturation has been well established^22, 23^, current evidence is premature to conclude on their role regulating DC phenotype and function. Nevertheless, previous *in vitro* studies have highlighted the importance of culture conditions favouring cell-to-cell contact for the induction of human NK cells with regulatory functions (i.e., IL-10 producing, HLA-G^+^ NK cells)^24^. In this context, it is tempting to speculate that a similar mechanism may be involved in the upregulation of the IL-10^+^ NK cell subset observed upon FLT3 treatment in the present study. Collectively, our findings suggest that an intact IL-10 axis ensures a successful interaction of NK cells with DC and may be important for fetal survival.

Our experiments using syngeneic matings also demonstrated that such an innate DC-NK cell liaison influences later developmental processes, especially placentation and fetal growth. The adoptive transfer of IL-10^+/+^ NK cells into a DC expanded IL-10^−/−^ gravid mice was sufficient to improve fetal weight and development in later gestation. The role of IL-10 in angiogenesis associated with gestation has been previously demonstrated in our lab. In particular, we have shown that exposure to environmental toxicants such as polychlorinated biphenyls (PCBs) result in preterm birth, intrauterine growth restriction and two-fold increase in amniotic fluid volume only in IL-10^−/−^ pregnant mice^25^. At the maternal-placental interface, impaired spiral artery remodeling and placental angiogenesis were observed. Now, we provide new evidence that IL-10 is also able to improve fetal growth restriction (FGR) caused by the FLT3 treatment in IL-10^−/−^ gravid mice. The protective role of IL-10 derived from NK cells is evidenced by the optimization of the spiral artery remodeling process and the rescued FGR observed in offspring carried by IL-10^−/−^ mice. In connection with this data, in an experimental LPS rat model of fetal demise and growth restriction, IL-10 has also been shown to attenuate LPS-induced fetal death rate and FGR^26^.

Taken together, our results provide direct evidence that the capacity of NK cells to secrete IL-10 is required to maintain the successful “innate” modulation of DC phenotype in the gravid uterus. This initial conversation between NK and DC cells is able to influence later developmental processes, especially placentation and fetal growth. Further elucidating the fundamental biology of this unique crosstalk will undoubtedly provide insights on how decidual cells maintain immune and angiogenesis homeostasis during early gestation. In addition, IL10 deficiency could contribute to human gestational disorders in which an enhanced inflammatory milieu and altered DC cell responses are implicated, such as spontaneous abortion and preeclampsia.

## Methods

### Mice

C57BL/6, Balb/c, IL-10^+/+^ and IL-10^−/−^ mice were purchased from Charles River Laboratories (Germany) and maintained in our animal facility with a 12 L/12D cycle. After cohabitation with males, the presence of a vaginal plug in females was designated as embryonic day (ED) 0.5. All of the animal procedures were approved by the Ethics Committee of Charité-Universitätsmedizin Berlin and conducted in accordance with the Landesamt für Gesundheit und Soziales (LAGeSo, license G0208/10) guidelines for humane treatment of laboratory animals. On ED7.5 and 13.5 mice from the respective groups (n = 6–8) were anaesthetized, and blood was obtained by retro-orbital puncture. The mice were then sacrificed and uterine tissue from the implantation sites was processed for histological sectioning and isolation of total protein and RNA according to standard procedures. Of note, only viable implantation sites were included when samples were collected for analysis.

### Fms-related tyrosine kinase 3 ligand (FLT3) Treatment

C57BL/6 and IL-10^−/−^ female mice with vaginal plugs were treated i.p. with human recombinant FLT3 (BE0098, BioX cell, 10 μg/mouse/day) for 7 consecutive days to expand DC. Combined DC expansion and NK cell depletion was achieved in FLT3 treated female mice by injecting anti-asialo GM1 Ab (986–10001, WAKO, 2 μg/g BW) on ED4.5 at the same concentration as described above.

### *In vivo* treatments

To test the effect of IL-10 on NK depletion, ED4.5 FLT3 treated female mice were injected i.p. with a cocktail of anti-asialo GM1 Ab and recombinant mouse IL-10 (14-8101-62, eBioscience 25ng/g BW) then sacrificed on ED7.5 or ED13.5 as described above. On ED5.5, dams received a second IL-10 injection. To further characterize the role of IL-10 in semiallogeneic pregnancies, Balb/c males were mated with either C57BL/6 IL-10^+/+^ or C57BL/6 IL-10^−/−^ female mice. Starting at ED0.5, dams received daily i.p. injections of human recombinant FLT3 (10 μg/mouse/day) for 7 consecutive days to expand DC. Combined DC expansion and NK cell depletion was achieved in FLT3 treated female mice by injecting anti-asialo GM1 Ab on ED4.5 as described above. In control animals, anti-asialo GM1 was replaced by normal rabbit serum at the same concentration.

### Adoptive transfer experiment

Uterine cells from FLT3 treated IL-10^+/+^ dams were isolated on ED 5.5 as described before^27^, stained with anti-NK1.1-PE and selected using a FACS Aria cell sorter (BD Biosciences). More than 95% pure NK1.1^+^ cells were obtained. Before injection, cells were counted by trypan blue exclusion. IL-10^−/−^ dams received an intravenous injection of 10^5^ NK1.1^+^IL-10^+/+^ cells or 10^5^ NK1.1^+^IL-10^−/−^ cells 24 h post FLT3 treatment, then sacrificed on ED13.5 (n = 6–7). On ED13.5, fetuses were fixed with Bouin’s solution for 2 to 24 h, washed with 70% ethanol several times (daily, until Bouin was cleared) and stored in 70% ethanol at room temperature. Fetal body weight was recorded after fixation. Developmental stages were determined as previously described according to Theiler Stage (TS) criteria (http://www.emouseatlas.org/emap/home.html).

### *In vitro* stimulation of decidual DC

On ED7.5 decidual DC were purified from uterine tissue of Balb/c mated C57BL/6 female mice as previously described^27^ and stimulated with lipopolysaccharide (LPS; 1 μg/ml) in the presence of recombinant IL-10 (14-8101-62, eBioscience, 10ng/ml) or anti-IL-10-receptor antibody (1B13a, BD Biosciences, 10 μg/ml) for 24 h. Of note, ED 7.5 was chosen for cell enrichment experiments since during this particular time the phenotype of DC is critical for pregnancy maintenance^28^. In addition, decidual DC were purified from IL-10^−/−^ dams, co-cultured in presence of NK1.1^+^IL-10^+^ cells and then stimulated with LPS and anti-IL-10-receptor antibody for 24 h as described above. The cells from both experiments were stained following our standard protocol^27^. I-A^b^ (553550, BD Bioscience) and CD86 (PM-IM2853, Immunotech) expression were determined by flow cytometry on a FACSCalibur analyzer (Becton Dickinson). FlowJo software was used for data analysis. Flow cytometry results were expressed as the percentage of cells positive for the surface markers evaluated.

### Enzyme-Linked Immunosorbent Assay (ELISA)

Serum samples from ED7.5 and 13.5 were tested in competitive ELISA using the mouse soluble Flt-1 (sFlt-1) Quantikine Immunoassay (R&D Systems, MVR100) following the manufacturer’s recommendations.

### RNA isolation and qRT-PCR

Total RNA isolation on E7.5 and E13.5, subsequent cDNA synthesis, and quantitative real time PCR were performed using our standard protocol^29^. Primer sequences are IL-10 (forward primer: 5′-GGTTGCCAAGCCTTATCGGA-3′, reverse primer: 5′-ACCTGCTCCACTGCCTTGCT-3′), VEGF (forward primer: 5′-ATCTTCAAGCCGTCCTGTGT-3′, reverse primer: 5′-GCATTCACATCTGCTGTGCT-3′), Flt-1 (forward primer: 5′-CGGAAGGAAGACAGCTCATC-3′, reverse primer: 5′-CTTCACGCGACAGGTGTAGA-3′) and HPRT (forward primer: 5′-GTTGGATACAGGCCAGACTTTGT-3′ and reverse primer 5′-CACAGGACTAGAACACCTGC-3′).

### Placenta histological analysis

Paraffin embedded uterine samples (whole implantation sites containing decidua, embryo and placenta) from E7.5 or ED13.5 were cut in 4 µm thick sections and stained with Masson’s trichrome, DBA lectin/Periodic acid-Schiff (PAS) and Isolectin B4 according to standard procedures^20^. Details are available in SI. Tissue sections were examined using a light microscope (Axiophot) and photographs taken with an Axio Cam HRc. Photo documentation was performed using the digital image analysis system Spot advanced software, version 8.6 (Visitron Systems).

### Statistics

The number of animals included in each experimental group was indicated accordingly. Data are presented as mean ± SEM from two or three replicate experiments. We analyzed mouse data by nonparametric Mann-Whitney U-test and ANOVA. Differences among groups were evaluated using the *t*-test or Tukey’s test respectively. We considered a p-value less than 0.05 as statistically significant as analyzed by GraphPad Prism 7.0 (GraphPad Software, Inc.).
